# Autologous Stem Cell Transplant for HIV-Associated Lymphoma: A Systematic Review and Meta-Analysis

**DOI:** 10.3390/cancers18152373

**Published:** 2026-07-23

**Authors:** Maria F. Comelles, Alexandra Grudzinski, Lisa K. Hicks

**Affiliations:** 1 Translational Medicine Graduate Program, Queen’s University, Kingston, ON K7L 3N6, Canada; 2 Michael G. DeGroote School of Medicine, McMaster University, Hamilton, ON L8S 1C7, Canada; 3 Division of Hematology/Oncology, St. Michael’s Hospital, Toronto, ON M5B 1W8, Canada; 4 Department of Medicine, University of Toronto, Toronto, ON M5B 1W8, Canada

**Keywords:** HIV infections, lymphoma, stem cell transplantation, autologous, antiretroviral therapy

## Abstract

Background: Despite improved outcomes with antiretroviral therapy, HIV-associated lymphoma (HAL) remains a major cause of mortality. Autologous stem cell transplant (ASCT) may be curative for relapsed/refractory (R/R) HAL, but HIV-related immunosuppression complicates management. We systematically reviewed prospective evidence on ASCT efficacy and toxicity in HAL. Methods: PubMed, Cochrane, Embase, and ClinicalTrials.gov were searched for publications between 1 January 1996 and 21 June 2026, with an initial search in January 2024 updated in June 2026. Non-English studies, retrospective analyses, studies with fewer than 10 eligible patients, and studies of primary CNS lymphoma were excluded. Studies of first-line ASCT were included for toxicity analysis but excluded from efficacy analysis. Analyses were performed using MedCalc v22.032. Results: 378 titles were screened, 350 underwent abstract review and 40 full-text review. Six non-randomized prospective studies were identified. Identified trials included a total of 134 patients (33 Hodgkin lymphoma, 101 non-Hodgkin lymphoma). Median age and CD4 counts reported by individual studies ranged from 39 to 47 years and 172–279 cells/µL, respectively; 94.2% of patients were male. All patients received antiretroviral therapy at time of ASCT. Pooled six-month non-relapse mortality (NRM) was 5.39% (95% CI: 2.28–9.73). Among the 52 R/R HAL patients with survival data available, estimated 2-year overall survival (OS) and progression-free survival (PFS) were 79.8% (95% CI: 68.1–89.3) and 77.9% (95% CI: 65.9–87.8), respectively. Conclusions: In this first systematic review and meta-analysis of ASCT for HAL, NRM post-ASCT is consistent with historical trial results in the non-HIV population. OS and PFS are encouraging, but prospective data are limited and comparative data is absent.

## Simple Summary

People with human immunodeficiency virus (HIV) are living longer thanks to contemporary antiviral treatment, but lymphoma remains a serious and potentially life-threatening complication. Autologous stem cell transplant, a procedure in which a patient’s own stem cells are used after high-dose chemotherapy, can cure some patients with relapsed aggressive lymphoma. However, because HIV affects the immune system, there have been concerns about the safety and effectiveness of this approach in this population. We conducted a systematic review to summarize information from existing prospective studies on the use of autologous stem cell transplant in people living with HIV-associated lymphoma. Our findings suggest that treatment-related mortality is low and survival outcomes are encouraging, although high-quality comparative trials are lacking. This work highlights the need for more rigorous studies and may help guide clinicians and researchers in improving care for this underserved population.

## 1. Introduction

HIV-associated lymphomas (HAL) are types of lymphoma that develop in people living with HIV (PLWH) [1], who remain at substantially increased risk of cancer despite advances in antiretroviral (ART) therapy [2,3]. HIV is estimated to account for 6.9% of incident non-Hodgkin lymphoma (NHL) and 6.1% of Hodgkin lymphoma (HL) cases globally, with the greatest burden occurring in Africa. In 2022, HIV was also estimated to be responsible for 12,800 NHL cases worldwide, contributing to an overall 81,300 HIV-attributable cancer cases, highlighting their persistent global burden [4,5,6]. Also, NHL and HL still remain among the leading causes of death among PLWH [7] and have a more aggressive course with advanced disease, extranodal involvement, and poor prognosis [8]. As cancer remains a leading cause of death among PLWH, rigorous evaluation of treatment outcomes is essential to optimize care and improve survival.

The Parma Group trial and subsequent clinical studies established autologous stem cell transplant (ASCT) as the standard of care for patients with chemotherapy-sensitive, relapsed/refractory (R/R) NHL and HL [9], but HIV infection has historically been regarded as a contraindication to ASCT. In the pre-ART era, PLWH were largely excluded from intensive chemotherapy regimens and ASCT because of concerns regarding uncontrolled HIV replication, profound immunosuppression, opportunistic infections, baseline marrow dysfunction, and poor treatment outcomes [10]. However, the introduction of ART in 1996 led to improved viral suppression and immune reconstitution, enabling PLWH to tolerate more aggressive therapies [11]. Subsequently, ASCT became a treatment option for chemo-sensitive R/R HAL. Molina [12] and Gabarre et al. [13] were among the first to advocate for more dose-intensive therapy in a selected group of HAL-NHL patients around the 2000s. Subsequently, research groups in Europe and the United States began to prospectively study this topic. Over the past two decades, it has been reported that HAL patients undergoing ASCT can achieve outcomes similar to those of HIV-negative individuals [14,15,16]. However, despite this shift in clinical practice, the historical exclusion of PWH from ASCT clinical trials has created persistent disparities in the evidence base and treatment patterns, limiting high-quality data to guide transplant decision-making in this population [17]. Additionally, most available evidence comes from specialized centers and heterogeneous studies, relying on non-comparative analyses or registry-based comparisons [18,19]. Consequently, current guidelines encourage the inclusion of PLWH in cancer clinical trials whenever appropriate, recognizing that their participation is essential for generating evidence to inform safe and effective cancer care in this population [20]. Careful patient selection and advances in supportive care for PLWH have led to encouraging long-term outcomes, with 2- and 5-year overall survival (OS) rates after ASCT in small, single-center studies [16,21]. Based on these results, clinicians consider ASCT as a reasonable therapeutic option in this population, but the toxicity and efficacy of ASCT have never been systematically reviewed for HAL.

The primary objective of this systematic review and meta-analysis is to evaluate the safety of ASCT in PLWH with aggressive HAL by systematically reviewing the available prospective evidence and performing a meta-analysis of NRM where feasible. The secondary objective is to evaluate the efficacy of ASCT in PLWH with R/R HAL by systematically reviewing OS and PFS following ASCT and performing meta-analyses of OS and PFS where sufficient homogeneous data are available.

## 2. Materials and Methods

The systematic review and meta-analysis are reported according to the 2020 Preferred Reporting Items for Systematic Reviews and Meta-Analyses (PRISMA) guidelines [22]. See PRISMA checklist in Supplementary Materials (Table S1).

### 2.1. Identification and Selection of Articles

PubMed, Cochrane, Embase, and ClinicalTrials.gov were searched between 1 January 1996 and 21 June 2026. The initial systematic search was conducted in January 2024. Given the time elapsed since the initial search, the same strategy was re-run on 21 June 2026, to identify additional eligible studies published in the interim. The following combined Medical Subject Headings (MeSH) and keywords were used: (HIV OR AIDS) AND (Lymphoma OR Hodgkin) AND (Peripheral Blood Stem Cell Transplant OR Autologous Stem Cell Transplant OR Allogeneic Stem Cell Transplant OR Bone Marrow Transplant OR Transplantation, Autologous) AND (Lymphoma, AIDS-Related/therapy). No additional studies met inclusion criteria in the updated search. The year 1996 was set as the initial year for the search because this year was the beginning of the era of highly active ART, including the combination of proteasome inhibitors and nucleoside reverse transcriptase inhibitors [23].

All identified studies were uploaded into the Covidence systematic review management software (Veritas Health Innovation, Melbourne, Australia, 2024). Studies were included if they were written in the English language, were prospective, and reported outcomes in more than 10 eligible patients. Retrospective studies were excluded. We also excluded patients with solid tumors, primary central nervous system lymphoma, mixed disease types where the lymphoma cases were less than 10 and/or could not be separately extracted, cases of patients less than 18 years old and expert review/opinions. Studies and sub-studies of first-line ASCT were included in the analysis of toxicity and excluded from the analysis of efficacy.

The information extracted included demographics, CD4 count, viral load, ART use, disease type, NRM, OS at two years, and PFS at two years. Title, abstract screening and full-text review was performed by two authors (MFC and AG) with a third author (LKH) available to resolve any discrepancies through consensus. Data extraction was performed by two authors independently (MFC and AG) to reduce the risk of bias and minimize errors.

### 2.2. Study Outcomes

The primary outcome of this study is NRM following ASCT, defined as death from any cause other than disease relapse occurring within 12 months after ASCT. NRM analyses included R/R HAL patients and HAL undergoing ASCT after any line of therapy. Secondary outcomes include OS and PFS at two years after ASCT for R/R HAL, restricted to studies reporting comparable 2-year OS and PFS estimates in cohorts with more than 10 patients. This distinction reflects that transplant-related toxicity is primarily a function of peri-ASCT management and patient factors (such as age and CD4 count) and is expected to be comparable regardless of disease status at ASCT. In contrast, survival outcomes differ substantially by disease status: patients transplanted in the first complete remission (consolidative intent) have a fundamentally more favorable prognosis than those transplanted for R/R disease (salvage intent), such that pooling these populations for efficacy would confound the estimate with a healthier, lower-risk subgroup. PFS was defined as the number of patients who did not suffer from death, disease relapse, or progression at two years from the day of ASCT. OS was defined as the number of patients who did not suffer from death at two years from the day of ASCT.

### 2.3. Risk of Bias Assessment

Risk of bias was assessed using the Joanna Briggs Institute (JBI) checklist for quasi-experimental studies [24].

### 2.4. Effect Measures

Effect measures were prespecified; however, minor modifications were made during the review to reflect the outcomes most consistently reported across the included studies. Specifically, time-to-event outcomes (OS and PFS) were summarized as survival proportions at the most frequently reported time point, which was two years after ASCT. When 2-year OS or PFS estimates were not explicitly reported in the text or tables, survival probabilities were extracted from the published Kaplan–Meier curves by visual estimation at the 2-year time point. A random-effects model was prespecified because the included studies were not expected to estimate a single common treatment effect owing to anticipated clinical and methodological differences between study populations and ASCT practices. Accordingly, the objective was to estimate the average treatment effect across a distribution of true study effects rather than assume one common underlying effect. Hazard ratios and other comparative effect measures were not synthesized due to a lack of comparative data.

### 2.5. Synthesis of Evidence

Studies were grouped for synthesis based on clinical and methodological characteristics, including patient population, intervention (prospective follow-up after ASCT), and reported outcomes. Due to heterogeneity in study design and outcome reporting, only outcomes with consistent definitions across studies (NRM and 2-year PFS and OS) were pooled. For the NRM synthesis, eligibility required prospective reporting of ASCT-related death within 12 months of ASCT, regardless of disease status (first-line or R/R) or histology, since NRM was assessed across the full transplanted cohort. For the OS/PFS synthesis, eligibility is restricted to R/R HAL patients with survival data reported at a comparable 2-year timepoint. Studies or sub-cohorts consisting predominantly of patients transplanted in first complete remission or studies in which eligible R/R outcomes cannot be extracted were excluded from this synthesis, as efficacy outcomes of consolidative ASCT in first remission are not comparable to salvage ASCT in the R/R setting. Similarly, studies including patients with T-cell lymphoma were eligible for the NRM analysis because NRM is less dependent on lymphoma histology. Efficacy analyses (OS and PFS) were restricted to studies reporting outcomes for patients with B-cell lymphoma treated after one to two lines of salvage therapy, as survival outcomes are not directly comparable between B-cell and T-cell lymphomas.

No substantial data transformations were required. Results of individual studies were summarized in structured tables, including study characteristics, patient populations, and reported outcomes. Where appropriate, outcomes were presented using descriptive statistics and tabulated comparisons. Graphical representations, such as forest plots, were generated when meta-analysis was feasible.

Meta-analysis of pooled results was completed using MedCalc Statistical Software version 22.032, where feasible. I2 tests were performed to assess for heterogeneity, where values of 25%, 50% and 75% correspond to low, moderate, and high levels of heterogeneity, respectively. The outcomes were described as incidence rates with 95% confidence intervals (CI). Meta-analysis was performed only for outcomes with sufficient homogeneity in definitions and reporting. Subgroup analyses and meta-regression were not performed because only six prospective studies were eligible overall, and only two contributed to the pooled OS/PFS analyses, making these analyses statistically unreliable. Sensitivity analyses were not performed because the limited number of eligible studies and small overall sample size would have yielded unstable pooled estimates with limited interpretability.

### 2.6. Reporting Bias Assessment

Assessment of publication bias was not performed due to the small number of included studies (<10), which limits the reliability and interpretability of funnel plot-based methods and statistical tests for asymmetry [25].

### 2.7. Registration and Protocol

This systematic review was not registered in a public registry; however, a formal review protocol was prepared prior to conducting the review. The review question, eligibility criteria, and search strategy were developed a priori and specified before study selection commenced. A draft search strategy was developed in advance and refined prior to implementation. The screening process, including the use of independent reviewers for study selection and predefined procedures for resolving disagreements, was also established a priori. Data extraction variables were prespecified before data collection; however, minor modifications were made during the review to accommodate additional variables or differences in reporting identified in the included studies. The critical appraisal tool was not prespecified, as the design of the eligible studies was not known at the time of protocol development. The risk of bias assessment tool was therefore selected after study inclusion, based on the methodological characteristics of the included studies, to ensure an appropriate appraisal approach.

## 3. Results

### 3.1. Study Selection

**Figure 1:**
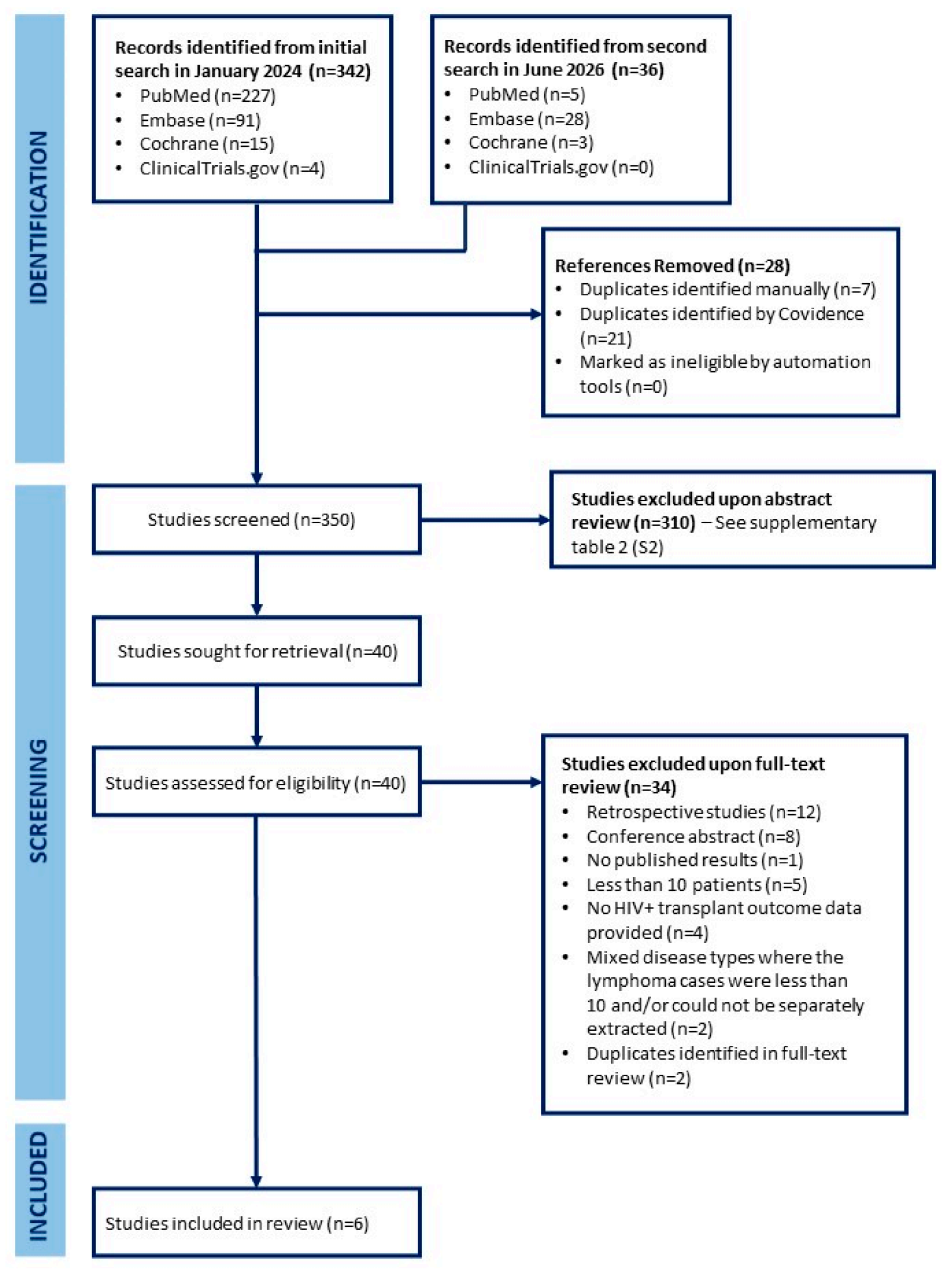
PRISMA 2020 flow diagram of study identification and selection (initial search: January 2024; updated search: June 2026). See supplementary Table S2 with reasons for exclusion of 310 studies upon abstract review.

A combined total of 378 records were identified: 342 from the initial search (January 2024) and 36 from the updated search (June 2026). Of the 36 records identified in the updated search, 1 was a duplicate. In total, 28 duplicate records were removed across both search rounds, leaving 350 unique records for abstract review. Of these, 310 were excluded upon abstract review due to the presence of retrospective studies, conference abstracts, results not published, mixed disease types where the lymphoma cases were less than 10 and/or could not be separately extracted, different population, or unrelated topic (Supplementary Table S2). Forty articles were identified for full-text review and 34 studies were excluded. Finally, six prospective studies were included and none were randomized controlled trials; see Figure 1.

### 3.2. Characteristics of Included Studies

Half of the studies were conducted in Europe and half in the United States. All of the studies were non-comparative prospective quasi-experimental studies. There were no randomized controlled trials. One study consisted of a letter to the editor publication [16]. Across the six prospective studies, 181 patients were enrolled, of whom 134 (74.0%) proceeded to ASCT: 33 with HL and 101 with NHL. Of the 87 patients for whom sex was reported, 82 (94.2%) were male and only five (5.8%) were female; two studies did not report sex distribution. Only two of the included studies reported participant race or ethnicity. The median age was comparable across studies (39.0–47.5 years), and the reported oldest transplanted patient was 68 years. NHL predominated in all studies, and BL was included in four studies. Small numbers of patients with T-cell lymphoma undergoing ASCT were included in three studies (Krishnan et al. [26], Re et al. 2018 [16], and Serrano et al. [14]), whereas the Alvarnas [27] et al. and Spitzer et al. [15] studies enrolled only patients with B-cell lymphoma. Re et al. 2009 [28] reported three patients with T-cell lymphoma, although it was not possible to determine whether these patients underwent ASCT. Patients with T-cell lymphoma contributed to the NRM analysis but were excluded from the pooled efficacy analyses. Median follow-up varied considerably, ranging from 6 to 50 months. Detailed information about demographics, sample size, and types of lymphoma included are in Table 1.

Baseline HIV status and infection prevention strategies were generally comparable across studies. Median CD4 counts ranged from 172 to 279 cells/μL, although individual patient values showed substantial variability. Most transplanted patients had suppressed or undetectable HIV viral loads before ASCT, and all studies incorporated ART as part of routine management. Infection prophylaxis was consistently administered, although antimicrobial regimens varied across studies according to institutional practice and local protocols (Table 2).

Eligibility criteria for ASCT were broadly consistent across studies. The majority of the studies required adequate organ function and excluded patients with active opportunistic infections. All but two studies specified age criteria for ASCT eligibility, and all permitted inclusion of older patients, up to 65 years in one study and up to 60 years in two studies, while one study did not define an upper age limit. However, HIV-specific eligibility criteria varied, particularly regarding minimum CD4 thresholds, ART requirements, viral suppression, and hepatitis B/C screening. Cardiac function thresholds also differed modestly between studies, whereas renal function and total bilirubin requirements were generally comparable. Detailed study-specific eligibility criteria are presented in Table 3.

The studies were generally at moderate risk of bias, which is expected for single-arm prospective ASCT studies. The most common limitation was the absence of a prospective control group. One study [27] incorporated a retrospective matched registry control. Another study [14] generated a control group from a single site but did not report on critical NRM or efficacy endpoints in the control group. This was considered an uncontrolled study with regard to the endpoints of interest. Strengths of the studies included prospective study design, clearly defined eligibility criteria, objective toxicity and survival endpoints, and longitudinal follow-up. Key limitations included small sample sizes, heterogeneity in conditioning regimens and supportive care practices, and early study closure due to slow accrual in one study. A JBI assessment for bias grade was conducted on all studies meeting the inclusion criteria; see Supplementary Table S3.

### 3.3. Non-Relapse Mortality

**Figure 2:**
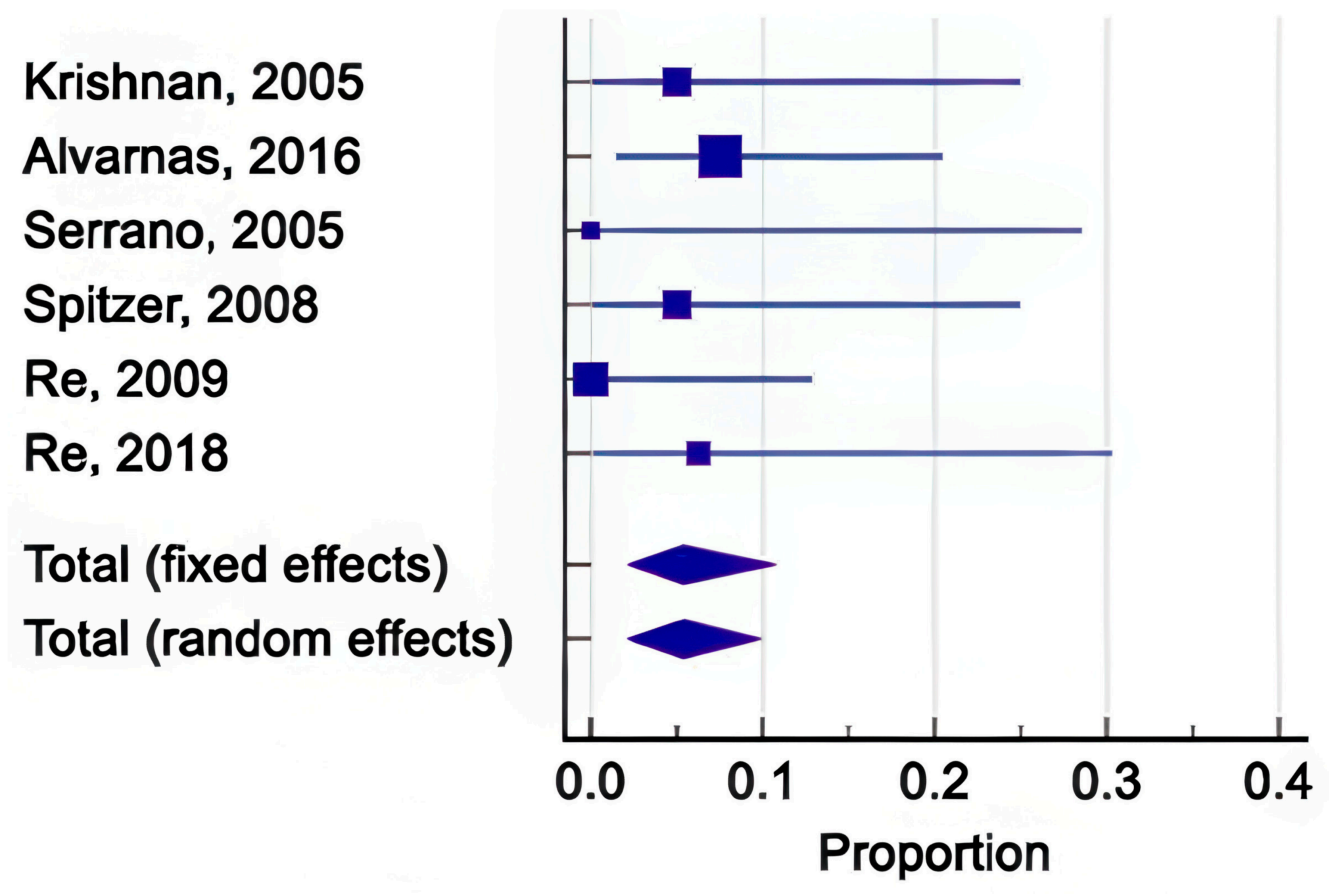
Forest plot of 12 months post-ASCT NRM in PLWH with aggressive HAL (*n* = 132). Analyses include both first-line and relapsed/refractory ASCT studies. Individual study estimates are presented as proportions with 95% CIs. Square sizes are proportional to the inverse-variance weight assigned to each study in the random-effects meta-analysis. Horizontal lines represent 95% CIs, and the diamond represents the pooled estimate with its 95% CI. CI: confidence interval. HAL: HIV-associated lymphoma. NRM: non-relapse mortality. ASCT: autologous stem cell transplant [14,15,16,26,27,28].

Six prospective studies met the predefined eligibility criteria for the safety analysis and were included in the NRM meta-analysis. Both first-line and R/R ASCT studies were eligible because NRM reflects ASCT-related toxicity rather than disease-specific efficacy. There was variability in the patient inclusion criteria across prospective studies in terms of response to salvage therapy and a range of lines of treatment prior to ASCT. We have not included NRM events post-salvage, as included studies focus on events post-ASCT. NRM was defined a priori as death from causes other than disease relapse within 12 months after ASCT. Although follow-up for NRM extends to 12 months, all reported NRM events occurred within the first six months after ASCT. One study reported a death event at 15 months post ASCT due to a gastrointestinal infection, which was not included in the pooled analysis as it did not meet the predefined NRM definition [14]. Consequently, the pooled 12-month estimate is identical to the pooled 6-month estimate: 5.39% (95% CI: 2.28–9.73) using a random effects model (I2 0%, *p* = 0.69) (Figure 2). The observed statistical heterogeneity (I^2^ = 0%) should be interpreted cautiously because only six studies were included, limiting the power of heterogeneity statistics to detect between-study variability. The causes of NRM were reported in five of six studies. The causes included bacterial and fungal infection, cardiotoxicity, liver toxicity (venous occlusive disease) and gastrointestinal infection (pathogenic agent was not clarified or unknown). Information regarding NRM and conditioning regimen for ASCT are described in Table 4.

Given the small number of studies (*n* = 6) contributing to this synthesis, formal assessment of reporting bias (e.g., funnel plot, Egger’s test) was not performed, as such methods are unreliable with fewer than 10 studies [25]. The possibility that unpublished studies with higher NRM exist cannot be excluded.

### 3.4. Overall Survival and Progression-Free Survival

**Figure 3:**
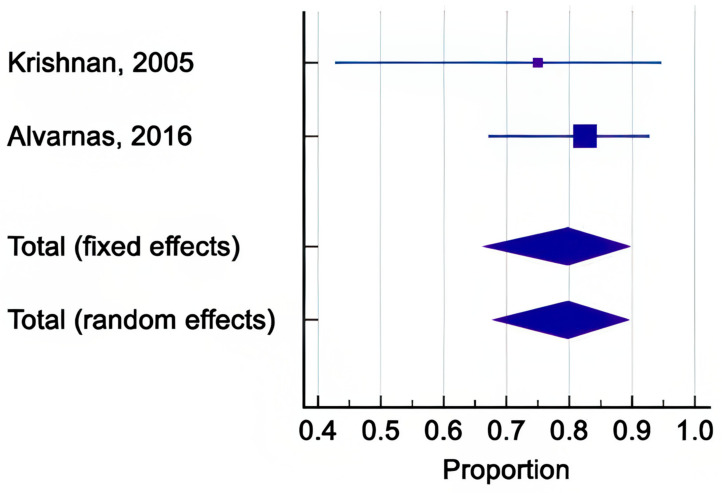
Forest plot of pooled 2-year OS following ASCT in patients with R/R HAL (*n* = 52). Individual study estimates are presented as proportions with 95% CIs. Square sizes are proportional to the inverse-variance weight assigned to each study in the random-effects meta-analysis. Horizontal lines represent 95% CIs, and the diamond represents the pooled estimate with its 95% CI. CI: confidence interval. HAL: HIV-associated lymphoma. OS: overall survival. R/R: relapsed/refractory. ASCT: autologous stem cell transplant [26,27].

**Figure 4:**
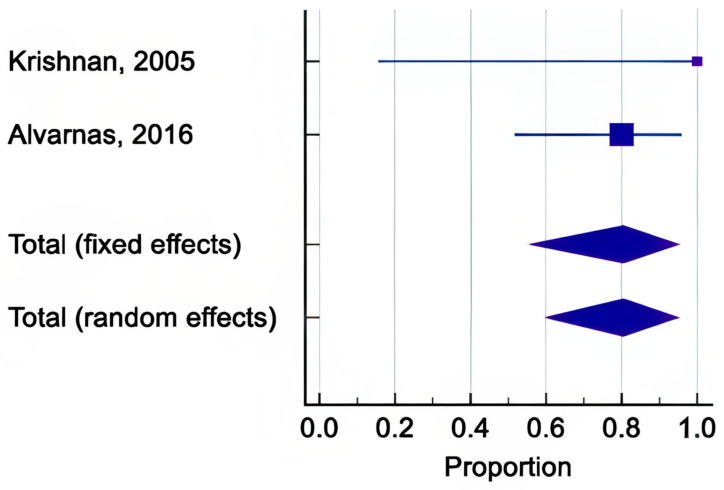
Forest plot of pooled 2-year OS following ASCT in patients with R/R HAL-HL (*n* = 15). Individual study estimates are presented as proportions with 95% CIs. Square sizes are proportional to the inverse-variance weight assigned to each study in the random-effects meta-analysis. Horizontal lines represent 95% CIs, and the diamond represents the pooled estimate with its 95% CI. CI: confidence interval. HL: Hodgkin lymphoma. HAL: HIV-related lymphoma. R/R: relapsed/refractory. OS: overall survival. ASCT: autologous stem cell transplant [26,27].

**Figure 5:**
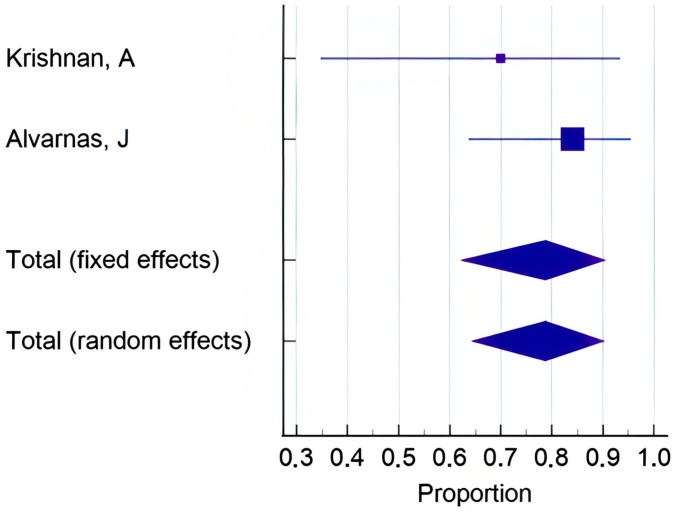
Forest plot of pooled 2-year OS following ASCT in patients with R/R HAL-NHL (*n* = 35). Individual study estimates are presented as proportions with 95% CIs. Square sizes are proportional to the inverse-variance weight assigned to each study in the random-effects meta-analysis. Horizontal lines represent 95% CIs, and the diamond represents the pooled estimate with its 95% CI. CI: confidence interval. NHL: non-Hodgkin lymphoma. HAL: HIV-related lymphoma. R/R: relapsed/refractory. OS: overall survival. ASCT: autologous stem cell transplant [26,27].

**Figure 6:**
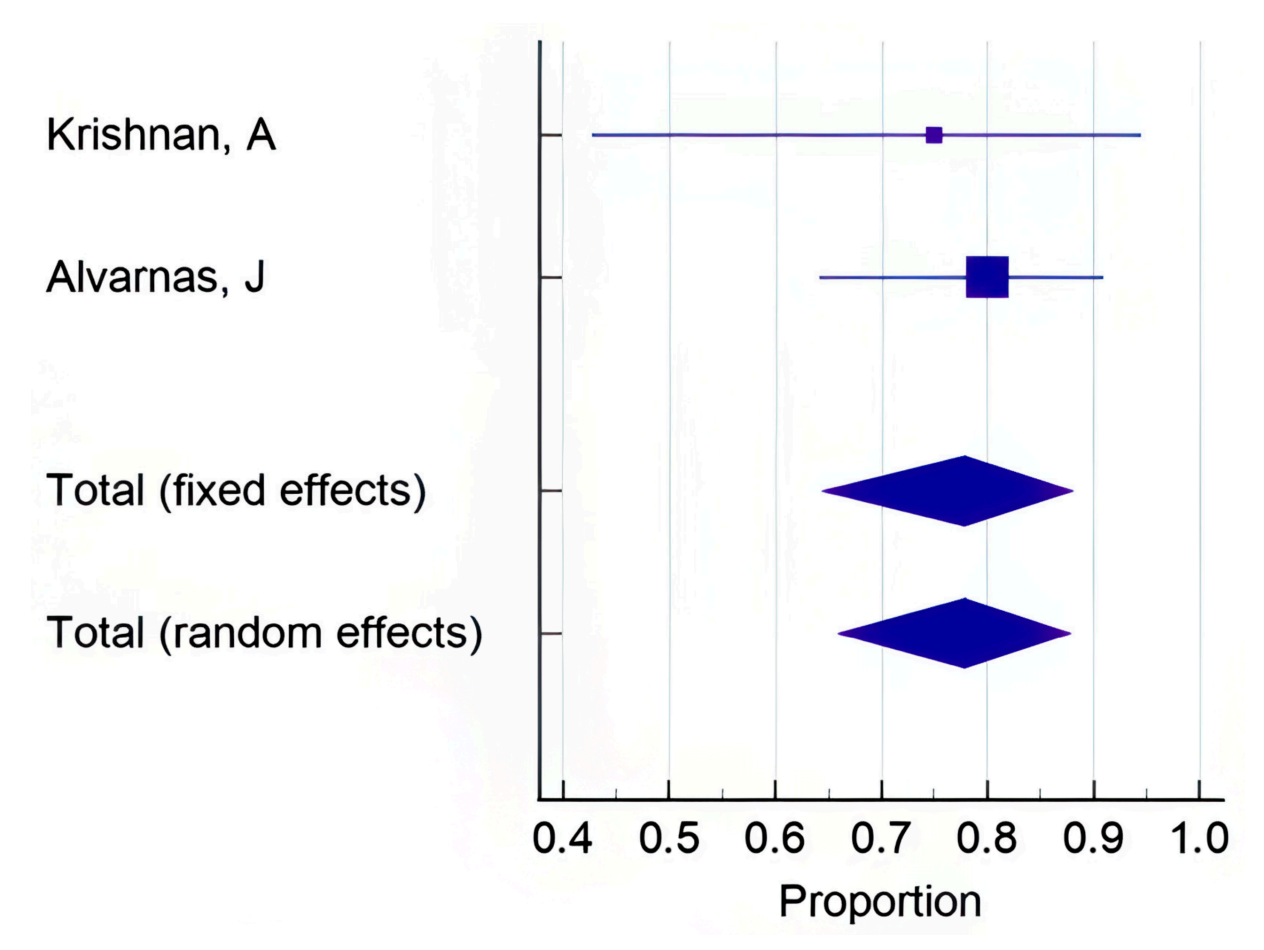
Forest plot of pooled 2-year PFS following ASCT in patients with R/R HIV-associated lymphoma (*n* = 52). Individual study estimates are presented as proportions with 95% CIs. Square sizes are proportional to the inverse-variance weight assigned to each study in the random-effects meta-analysis. Horizontal lines represent 95% CIs, and the diamond represents the pooled estimate with its 95% CI. CI: confidence interval. PFS: progression-free survival. R/R: relapsed/refractory. ASCT: autologous stem cell transplant [26,27].

**Figure 7:**
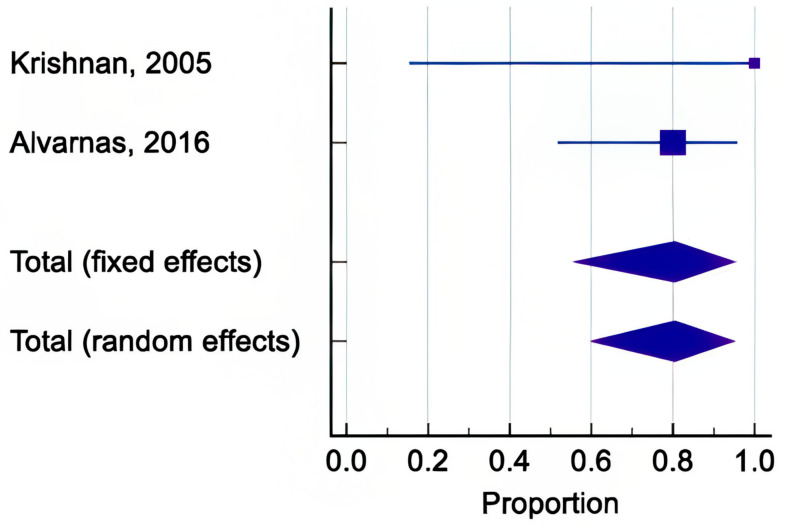
Forest plot of pooled 2-year PFS following ASCT in patients with R/R HAL-HL (*n* = 17). Individual study estimates are presented as proportions with 95% CIs. Square sizes are proportional to the inverse-variance weight assigned to each study in the random-effects meta-analysis. Horizontal lines represent 95% CIs, and the diamond represents the pooled estimate with its 95% CI. CI: confidence interval. HL: Hodgkin lymphoma. HAL: HIV-related lymphoma. PFS: progression-free survival. R/R: relapsed/refractory. ASCT: autologous stem cell transplant [26,27].

**Figure 8:**
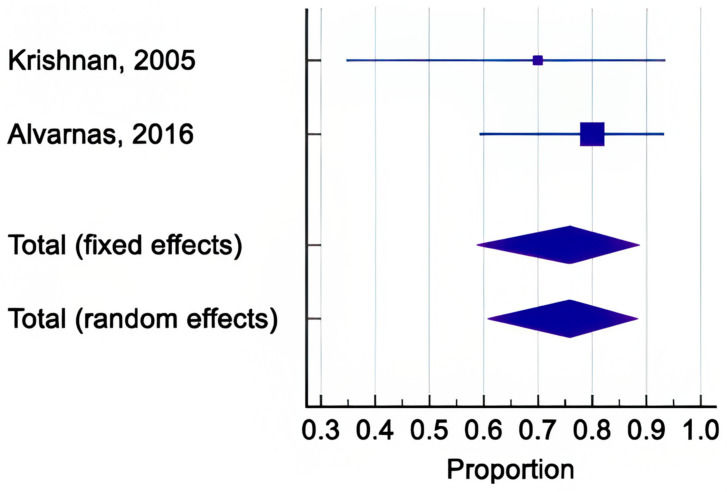
Forest plot of pooled 2-year PFS following ASCT in patients with R/R HAL-NHL (*n* = 35). Individual study estimates are presented as proportions with 95% CIs. Square sizes are proportional to the inverse-variance weight assigned to each study in the random-effects meta-analysis. Horizontal lines represent 95% CIs, and the diamond represents the pooled estimate with its 95% CI. CI: confidence interval. NHL: non-Hodgkin lymphoma. HAL: HIV-related lymphoma. PFS: progression-free survival. R/R: relapsed/refractory. ASCT: autologous stem cell transplant [26,27].

We excluded patients in first complete remission after first line of treatment from the efficacy analysis. We evaluated data from three unique prospective trials that provided OS and PFS estimates with variable follow-up after ASCT for R/R HAL: Alvarnas et al. [27], Krishnan et al. [26] and Serrano et al. [14]. Two studies, Alvarnas et al. [27] and Krishnan et al. [26], satisfy the predefined eligibility criteria for quantitative synthesis, providing comparable 2-year OS and PFS estimates in cohorts containing more than 10 eligible R/R HAL patients. We also meta-analyzed data for R/R HAL-HL and R/R HAL-NHL separately. However, the study performed by Serrano et Al. [14] could not be included in the meta-analysis for OS and PFS because fewer than 10 patients met the predefined eligibility criteria for the efficacy analysis (R/R HAL undergoing ASCT). A total of 52 patients from two studies were amenable to pooled analysis. The R/R HAL-HL synthesis pooled 17 patients (15 from Alvarnas et al. [27], 2 from Krishnan et al. [26]). The R/R HAL-NHL synthesis pooled 35 patients (25 from Alvarnas et al. [27], 10 from Krishnan et al. [26]). Risk of bias for both histology-specific syntheses reflects the same two contributing studies described above: Alvarnas et al. [27] incorporated a matched external comparator cohort from the CIBMTR registry, while Krishnan et al. [26] was a single-institution series without any comparator group. Both were judged at moderate risk of bias overall using the JBI, with the additional limitation that small subgroup sizes, particularly the HL subgroup (*n* = 17), further reduce precision of these estimates. Estimated 2-year OS was 79.8% (95% CI: 68.1–89.3), 80.5% (95% CI: 60.2–94.7), and 78.7% (95% CI: 63.3–90.2) for all R/R HAL, R/R HAL-HL, and R/R HAL-NHL, respectively. Estimated 2-year PFS was 77.9% (95% CI: 65.9–87.8), 80.5% (95% CI: 60.2–94.7), and 75.9% (95% CI: 61.0–88.1) for all R/R HAL, R/R HAL-HL and R/R HAL-NHL respectively. The I2 statistic for heterogeneity was 0% for OS/PFS but is not meaningful given that only two studies contributed to these endpoints. Given the small number of studies contributing to each pooled estimate, no further investigations of heterogeneity (e.g., sensitivity analysis or meta-regression) were conducted. Reporting bias could not be formally assessed for the OS/PFS synthesis given the very small number of contributing studies (*n* = 2) [25]. This synthesis is particularly vulnerable to the potential existence of unpublished or unreported trials with less favorable outcomes, and pooled estimates should be interpreted with this limitation in mind. Forest plot diagrams for OS are available in Figure 3, Figure 4 and Figure 5. Forest plot diagrams for PFS are available in Figure 6, Figure 7 and Figure 8.

## 4. Discussion

This is the first systematic review and meta-analysis in the ART era to evaluate NRM in HAL after ASCT and efficacy in R/R HAL after ASCT. Baseline characteristics are essential for assessing the generalizability of study findings and for determining the extent to which results can be extrapolated to patient populations outside clinical trials. In the prospective, non-randomized studies included in this systematic review, female patients were poorly represented, accounting for only 5.8% of the evaluable transplanted patients (5/87). Only two studies [15,27] reported data on patient race and/or ethnicity. In both, the representation of diverse populations was limited to White, Black, and Hispanic/Latino groups, with no further granularity. This is particularly relevant in the context of HIV-associated malignancies, where ethnic disparities and access to care are well documented [29]. Future prospective studies should adopt standardized reporting of participant demographics, including sex and race/ethnicity, and prioritize recruitment strategies that improve the representation of women and underrepresented PLWH. More representative study populations are needed to better inform clinical decision-making across diverse patient groups. However, we acknowledge that analyses of these subgroups within small cohorts may lack statistical power and may not reliably reflect true outcomes. The patient cohorts were consistently young across studies, as reflected in the baseline characteristics. The median age reported across individual included trials ranged from 39 to 47 years, with the oldest patient being 68 years. The included studies generally applied broad age eligibility criteria, allowing enrollment of older adults considered fit for ASCT, suggesting that chronological age alone was not an absolute barrier to ASCT. This is broadly consistent with real-world eligibility for ASCT (Table 3), which typically extends to approximately 65 years in fit individuals. Chronological age alone should not be the primary determinant of ASCT eligibility, as multiple studies have demonstrated that carefully selected, fit older patients can derive comparable benefit from ASCT across different disease settings [30,31]. As PLWH experience improved survival with ART, an increasing number of older patients will likely be considered for inclusion in ASCT studies, as well as in trials evaluating novel therapies. 

The pooled incidence rate of 5.39% for NRM within 12 months aligns with historical data for non-HIV patients, where NRM ranges from 5% to 10% over varying periods (six to 12 months, depending on the study) [32,33]. There were no events meeting NRM criteria between six to 12 months after ASCT, consistent with the higher risk of early death after this procedure. However, a recent systematic review analyzing outcomes after ASCT in ambulatory settings for non-HIV patients reported lower NRM rates, ranging from 0.5% to 4% at 100 days [34]. Moreover, the causes of NRM in our study align with previous reports, where bacterial infections are the primary early complications, while herpes zoster and CMV reactivation are the most common late complications. Consistently, three of the six patients that died within six months died of bacterial or fungal infection. It is important to note that the studies included in this meta-analysis employed heterogeneous inclusion criteria with respect to CD4 counts (ranging from 25 to 1064 cell/μL) and viral load (some trials allowing detectable viral loads). Some included patients had CD4 counts substantially lower than recommended by current guidelines for inclusion of PLWH in clinical trials [20]. Additionally, only three studies explicitly aligned with guidance by including patients with undetectable viral loads [14,26,27], whereas the remaining three did not specify or enforce this criterion. Given the lack of standardized inclusion criteria and the variability in baseline immune and virologic status, the results of this analysis should be interpreted with caution. Also, this meta-analysis does not account for NRM following salvage therapy, as all of the studies included in this analysis are focused primarily on ASCT outcomes; thus, the NRM in this meta-analysis likely underestimates the real-world NRM. On the other hand, all prospective studies were aligned with contemporary standards of care regarding ART, as all enrolled patients were receiving ART at the time of ASCT or shortly thereafter, aligning with modern recommendations.

Among the 52 assessable patients, the two-year OS was 79.8% for all R/R HAL, which compares favorably with previously reported outcomes for non-HAL patients undergoing ASCT, reporting approximately 70% OS at two years [35]. In the R/R HAL-HL subgroup, two-year OS was 80.5%, an encouraging result consistent with reports from other groups, demonstrating 75% at two years, in the non-HIV R/R HL setting using preparative regimens such as FTBI-based or BCNU-containing conditioning [36]. Our pooled OS estimate for R/R HAL-NHL, based on studies by Alvarnas and Krishnan, was 78.7% at two years. This is encouraging given the fact that in non-HIV R/R NHL, such as the PARMA study [9], a two-year OS of 70% was reported, although it was conducted in the pre-rituximab era using DHAP followed by radiation or BCNU-based conditioning, a similar approach to the study conducted by Krishnan; instead, Alvarnas used BEAM conditioning. Most rituximab-era series report longer-term outcomes, but Akhtar et al. provide a two-year benchmark of 63.8% OS in a mixed R/R cohort [37].

For the same cohort, PFS at two years was 77.9% for all comers in this analysis. A study by Nademanee et al. reported that for non-HIV R/R HL, the cumulative probability of 2-year PFS in 85 patients after ASCT was 58% [38]. In contrast, for R/R HAL-HL, our meta-analysis estimated a PFS of 80.5% at two years, significantly higher than the 51% PFS at two years reported for non-HIV HL for the placebo arm in the phase III AETHERA trial [35]. What is more, our meta-analysis reported a PFS of 75.9% (95% CI: 61.0–88.1) for R/R HAL-NHL. Interestingly, a registry-based, retrospective, individual-matched, case–control study by Díaz-Martín et al. [39] compared outcomes based on HIV serologic status and reported PFS for HIV HAL-NHL at 57.5% and 49.2% for control NHL with a median follow-up of 30 months. However, this study is retrospective, and subgroup analysis of patients with NHL and HL in first and second remission was not performed, so direct comparisons must be interpreted in the context of these limitations. All comparisons with historical HIV-negative ASCT cohorts provide useful clinical context but should be interpreted cautiously. We did not perform a formal quantitative comparison, and differences in patient selection, lymphoma subtype, ASCT era, conditioning regimens, supportive care, and study design limit the validity of indirect comparisons.

Importantly, there were seven patients with Burkitt’s lymphoma (BL) and two patients with plasmablastic lymphoma (PBL) within the cohort of patients reported by Alvarnas et al. [27] and four patients with BL in the cohort provided by Krishnan et al. [26], with no PBL patients in this last cohort. It is worth noting that the PFS and OS outcomes observed in this meta-analysis are reassuring, despite the inclusion of patients with high-risk histology such as BL and PBL, strongly associated with aggressive clinical behavior and historically poor prognoses at relapse [40,41]. Their inclusion could potentially lower the pooled survival estimates due to their inherently more aggressive disease course and limited therapeutic responsiveness. Additionally, the two studies included in the efficacy meta-analysis enrolled patients who had received one to two lines of salvage therapy to achieve a second complete remission before undergoing ASCT, introducing some heterogeneity in treatment exposure that may have influenced outcomes. Nevertheless, the favorable survival outcomes observed suggest that ASCT remains a beneficial approach, even within a heterogeneous patient population that includes these high-risk entities. Due to the small sample size, our analysis could not conduct subgroup analyses for these specific diseases or other factors that could impact efficacy, such as IPI score, age, or presence of other comorbidities. It is also important to note that efficacy analyses were restricted to patients with B-cell lymphoma because survival outcomes are not directly comparable between B-cell and T-cell lymphomas. Consequently, our pooled OS and PFS estimates should not be generalized to patients with T-cell lymphoma.

Although retrospective cohort studies have begun to explore the use of novel therapies in PLWH, such as chimeric antigen receptor T (CAR T)-cell therapy or bispecific antibodies, prospective data remain lacking in these settings. In particular, a recent retrospective analysis comparing outcomes of CAR T-cell therapy in patients with and without HIV demonstrated a significantly increased 1-year risk of infectious complications among PLWH, including sepsis (RR 1.75, 95% CI: 1.03–2.97) and bacteremia (RR 2.41, 95% CI: 1.41–4.12) [42]. These findings highlight important safety considerations in this population. However, generating prospective evidence and conducting subgroup analyses remains challenging, as PLWH are still frequently excluded from contemporary clinical trials [43,44]. This exclusion limits the availability of high-quality data and underscores the need for more inclusive trial designs to better characterize the safety and efficacy of these emerging treatments in this population.

Our study has important limitations. Our search was limited to four databases, which may have missed unpublished or non-indexed studies. This review intentionally restricted inclusion to prospective studies to provide the highest-quality available evidence and minimize the risk of selection bias, outcome ascertainment bias, and confounding commonly associated with retrospective studies. Although this approach reduced the number of studies available for quantitative synthesis, it strengthened the internal validity of the pooled estimates. Inclusion of retrospective studies would have increased the sample size but at the expense of substantially greater clinical and methodological heterogeneity, making interpretation of pooled estimates more challenging. For studies that did not report a discrete 2-year OS/PFS estimate in text, values were extracted directly from published Kaplan–Meier curves, which introduces a degree of measurement imprecision inherent to visual data extraction. Additionally, we were unable to formally conduct sensitivity analyses, limiting our ability to determine whether our findings are robust to these review-level constraints. The small number of eligible prospective studies also precluded subgroup analyses or meta-regression, making it difficult to evaluate the robustness of the pooled estimates. These analyses were not feasible for several reasons. First, patient-level data for many clinically relevant characteristics were not consistently reported across the included studies, obviating reliable subgroup analyses. Second, only six studies and two studies contributed to the pooled NRM and efficacy analyses, respectively. Further stratification of this already limited sample would have resulted in unstable and clinically uninterpretable estimates. We acknowledge that the absence of subgroup analyses limits the ability to explore treatment effects across clinically relevant patient subgroups and, therefore, to inform individualized clinical decision-making. This highlights an important avenue for future research.

The studies included in this analysis are heterogeneous in terms of salvage therapies administered and the number of lines of salvage therapy. Hence, the observed I^2^ values should be interpreted cautiously because the small number of included studies limits the ability of heterogeneity statistics to reliably detect between-study differences. As well, PFS and OS estimates should be interpreted cautiously, as the included studies reported outcomes only among patients who underwent ASCT, potentially introducing selection bias by excluding patients who failed to reach ASCT. Cause-specific patterns of NRM should be interpreted cautiously because of inconsistent reporting across studies. It should also be noted that there were no randomized trials and that the overall body of literature is very small. The absence of randomized controlled trials in this setting likely reflects the inherent challenges of designing such studies for HAL. Their relatively low incidence, combined with the clinical heterogeneity of this population—including variability in CD4 counts, viral suppression, comorbidities, and lymphoma subtypes—limits the feasibility of adequately powered randomized studies. In addition, concerns regarding drug–drug interactions with ART, increased risk of infectious complications, and the need for specialized multidisciplinary care further complicate trial design and execution. These barriers have contributed to the predominance of single-arm prospective studies and registry-based analyses, underscoring the need for international collaboration and pragmatic approaches to generate higher-quality and expedited evidence in this population.

## 5. Conclusions

This is the first systematic review and meta-analysis to summarize the safety and efficacy of ASCT in patients with HAL. Although no comparative trials are currently available, our findings revealed a 5.39% NRM, with all ASCT-related mortality occurring in the first 6 months and no events occurring between 6 and 12 months after ASCT. This is consistent with that reported in historical cohorts of HIV-negative populations. Moreover, OS and PFS outcomes appear promising for R/R HAL, even in the context of a heterogeneous patient population that includes individuals with high-risk subtypes such as BL and PBL. Nonetheless, these results must be interpreted with caution given the limited availability of prospective data, the absence of standardized inclusion criteria across studies, and the complete lack of comparative data. These findings underscore the need for well-designed prospective trials to better define the role of standard of care treatments or novel therapies in patients with HAL. Their inclusion, identification, and analysis as subgroup in clinical trials is also encouraged.

## Figures and Tables

**Table 1:** General characteristics of the studies included in the analysis.

First Author	Alvarnas, J [27]	Krishnan, A [26]	Re, A [16]	Re, A [28]	Serrano, D [14]	Spitzer, T [15]
**Year of publication**	2016	2005	2018	2009	2005	2008
**Location**	United States	United States	Italy	Italy	Spain	United States
**Sample size (*n*)**	43	20	27	50	14	27
**Proceeded to transplant (*n*)**	40	20	16	27	11	20
**Median age (years)**	46.9	44	47.5	39	39.5	42
**Male (*n*)**	35	N/A	16	N/A	11	20
**Hodgkin lymphoma (*n*)**	15	2	0	8	3	5
**Non-Hodgkin lymphoma (*n*)**	25	18	16	19	8	15
**DLBCL (*n*)**	16	12	12	N/A	5	12
**BL (*n*)**	7	6	N/A	N/A	2	3
**T-cell histology (*n*)**	0	1	1	N/A	1	0
**Median follow-up (months)**	24.8	31.8	50	44	30	6

N/A: Not available. DLBCL: Diffuse Large B-Cell Lymphoma. BL: Burkitt’s Lymphoma.

**Table 2:** HIV status and infection prevention in transplanted patients in the analyzed studies.

First Author	Alvarnas, J [27]	Krishnan, A [26]	Re, A [16]	Re, A [28]	Serrano, D [14]	Spitzer, T [15]
**Proceeded to transplant**	40	20	16	27	11	20
**Median CD4 count, cell/μL (range)**	249 (39–797)	175 (25–1064)	279 (95–558)	190 (88–545)	172 (33–364)	203 (53–574)
**Undetectable viral load, copies/mL**	32	17	8	22	11	N/A
**Started treatment on ART**	40 ^a^	20	16	27	11	20
**Use of antimicrobial prophylaxis**	According to institutional standards ^b^	TMS, quinolones, azoles and acyclovir	Conventional antimicrobial prophylaxis ^b^	TMS ^c^, azoles and acyclovir	TMS, quinolones, azoles and acyclovir	TMS and azoles

ART: Antiretroviral therapy. TMS: Trimetoprim-Sulfametoxazol. N/A: Not available. ^a^ Held from the time of initiation of the BEAM regimen and resumed at least 7 days after completion of the preparative regimen or following recovery from transplant-related gastrointestinal toxicities. ^b^ Not otherwise detailed. ^c^ Not after ASCT to avoid myelotoxicity.

**Table 3:** Inclusion criteria for ASCT in the analyzed studies.

Study	Age (Years)	LVEF (%)	Opportunistic Infections	Renal Function ^a^	Total Bilirubin ^a^	Hepatitis B and C	ART Required	CD4 (Cells/μL)	Viral Load (Copies/mL)
**Serrano [14]**	≤65	>50	Must be absent	Cr < 2 mg/dL	<2 mg/dL	N/S	Yes	N/S	Undetectable
**Krishnan [26]**	N/S	>50	Must be absent	CrCl ^b^ > 60 mL/minute	Liver function tests < 2 × normal	Allowed ^c^	Yes	>100 ^d^	Undetectable
**Alvarnas [27]**	>15	N/S	Must be absent	Adequate organ function	Adequate organ function	N/S	N/S	N/S	Undetectable
**Spitzer [15]**	N/S	≥45	Must be absent	Cr ≤ 2 mg/dL	≤2 mg/dL	N/S	N/S	>50	<110,000
**Re, A 2009 [28]**	≤60	>50	Must be absent	Cr ≤ 2 mg/dL	≤3 mg/dL	Allowed	Yes	>100	N/S
**Re, A 2018 [16]**	≤60	N/S	N/S	N/S	N/S	N/S	Yes	>50	N/S

LVEF: Left Ventricular Ejection Fraction. N/S: Not specified. ART: Antiretroviral therapy. Cr: Creatinine. ^a^ Original wording extracted from protocol. ^b^ 24 h creatinine clearance. ^c^ Not an exclusion criterion, but required further evaluation, including a liver biopsy. ^d^ Only for the first five patients enrolled, omitted for subsequent entries.

**Table 4:** Treatment exposure and NRM in the analyzed studies.

First Author	Alvarnas, J [27]	Krishnan, A [26]	Re, A [16]	Re, A [28]	Serrano, D [14]	Spitzer, T [15]
**Proceeded to transplant (*n*)**	40	20	16	27	11	20
**Median Follow-up (months)**	24.8	31.8	50	44	30	6
**Conditioning regimen**	BEAM	BCNU, VP-16, Cy for NHL or FTBI, VP-16, Cy for HL	BEAM	BEAM	BEAM or BEAC	Dose reduced BU and Cy
**Rituximab received prior ASCT for NHL (*n*)**	Allowed ^a^	9/18	16/16	Allowed ^a^	2/8	Unknown
**NRM ^b^ events post ASCT (*n*)**	3	1	1	0	1	1
**Cause of death**	Fungal infection, cardiac arrest and unknown cause.	Cardiomyopathy and renal failure.	Klebsiella pneumoniae sepsis.	N/AP	Gastrointestinal infection.	Hepatic VOD.
**Timing of death (days from ASCT)**	N/A	22	33	N/AP	450	33

NHL: Non-Hodgkin lymphoma. HL: Hodgkin lymphoma. NRM: Non-relapse mortality. BCNU: Carmustine. BEAM: BCNU 300 mg/m^2^, etoposide 800 mg/m^2^ total, cytarabine 800–1600 mg/m^2^ total, and melphalan 140 mg/m^2^. VP-16: Etoposide. Cy: Cyclophosphamide. FTBI: Fractionated total body irradiation. Dose reduced BU: Busulfan 11.2 mg/kg intravenously. ASCT: Autologous Stem Cell Transplant. N/A: Not available. N/AP: Not applicable. VOD: Veno-Occlusive Disease. ^a^ The exact number of patients who received rituximab prior to proceeding to ASCT is not reported. ^b^ Occurring within 1 year, otherwise not specified.

## Data Availability

All data supporting the findings of this systematic review and meta-analysis are available from the corresponding author upon reasonable request.

## Supplementary Materials

The following supporting information can be downloaded at: https://www.mdpi.com/article/10.3390/cancers18152373/s1, Table S1: PRISMA 2020 checklist; Table S2: Excluded studies upon abstract review (*n* = 310); Table S3: JBI critical appraisal of included quasi-experimental studies.
