# Supplementary material for: Autologous Stem Cell Transplant for HIV-Associated Lymphoma: A Systematic Review and Meta-Analysis
Source: Cancers (Basel). 2026 Jul 23;18(15):2373. doi: 10.3390/cancers18152373 (PMC13464129; doi:10.3390/cancers18152373)
Supplement: Supplementary file 1 [file cancers-18-02373-s001.zip › Table S2_Studies excluded table_HIV_ASCTJuly13th.pdf]

Table S2: Excluded Studies Upon Abstract Review (n=310)

Authors (Date)	Study Type	Reason for Exclusion
Woolfrey (2017)	Interventional	Unrelated to review question
Bachanova (2020)	Interventional	Study not limited to people with HIV
Sauter (2016)	Interventional	Unrelated to review question
Ferreri (2016)	Interventional	Primary CNS Lymphoma
Kassam (2017)	Interventional	Primary CNS Lymphoma
Ferreri (2019)	Interventional	Primary CNS Lymphoma
Vitolo (2019)	Interventional	Does not include people with HIV
Ferreri (2022)	Interventional	Primary CNS Lymphoma
Ferreri (2023)	Interventional	Primary CNS Lymphoma
Illerhaus (2023)	Interventional	Primary CNS Lymphoma
Scordo (2023)	Interventional	Unrelated to review question
Hamadani (2021)	Interventional	Unrelated to review question
Kuruvilla (2021)	Interventional	Unrelated to review question
Symons (2020)	Interventional	Includes children
Sauter (2017)	Interventional	Unrelated to review question
Ferreri (2016)	Interventional	Unrelated to review question
Ferreri (2016)	Interventional	Primary CNS Lymphoma
Makowski (2016)	Interventional	Did not report on our study outcomes
Simonetta (2016)	Retrospective	Unrelated to review question
Birndt (2016)	Prospective	Unrelated to review question
Dhakal (2016)	Retrospective	Disease other than lymphoma
Winterling (2016)	Poster Presentation	Unrelated to review question
Salar (2014)	Interventional	Unrelated to review question
Kasow (2012)	Interventional	Includes children
Chawla (2012)	Interventional	Unrelated to review question
Blennow (2010)	Prospective	Unrelated to review question
Steensma (2010)	Expert Review	Unrelated to review question

Tsimberidou (2009)	Systematic Review	Unrelated to review question
Couriel (2009)	Interventional	Unrelated to review question
Brannagan III (2009)	Expert Review	Unrelated to review question
Tamura (2009)	Prospective	Unrelated to review question
Ning (2008)	Interventional	Unrelated to review question
Summers (2007)	Expert Review	Unrelated to review question
Elad (2006)	Prospective	Unrelated to review question
Schulenburg (2005)	Retrospective	Did not report on our study outcomes
Cornelissen (2003)	Prospective	Mixed disease types
Current Drug Discovery*(2003)	Expert Review	Unrelated to review question
Eisen (2002)	Prospective	Unrelated to review question
Avery (2001)	Interventional	Unrelated to review question
Kruger (1997)	Retrospective	Unrelated to review question
Vannucchi (1996)	Interventional	Unrelated to review question
Slavin (1995)	Interventional	Unrelated to review question
Ganapathi (2022)	Case Report	Study design
Khatib (2020)	Retrospective	Primary CNS Lymphoma
Gupta (2020)	Retrospective	Less than 10 patients
Cwynarski (2020)	Interventional	Primary CNS Lymphoma
Shindiapina (2019)	Interventional	Did not report on our study outcomes
Ferreri (2019)	Interventional	Did not report on our study outcomes
Hickey (2019)	Retrospective	Less than 10 patients
Shah (2018)	Retrospective	Unrelated to review question
Dandachi (2018)	Retrospective	Primary CNS Lymphoma
Maleki (2018)	Case Report	Study design
Cantwell (2018)	Retrospective	Primary CNS Lymphoma
Arslan (2018)	Systematic Review	Study design
Ni (2017)	Retrospective	Disease other than lymphoma
Ferreri (2015)	Interventional	Primary CNS Lymphoma

Uldrick (2015)	Expert Opinion	Study design
Ibrahim (2015)	Retrospective	Mixed disease types
Khan (2014)	Retrospective	Primary CNS Lymphoma
Graczyk (2014)	Retrospective	Primary CNS Lymphoma
Ferreri (2014)	Interventional	Primary CNS Lymphoma
Ferreri (2014)	Interventional	Primary CNS Lymphoma
Noy (2013)	Retrospective	Intervention other than ASCT
Hentrich (2011)	Interventional	Intervention other than ASCT
Fanale (2010)	Retrospective	Less than 10 patients
Hentrich (2010)	Interventional	Intervention other than ASCT
Gabriel (2008)	Case Report	Study design
Hagiwara (2017)	Interventional	Less than 10 patients
O'Neill (2015)	Interventional	Less than 10 patients
Durante (2015)	Retrospective	Less than 10 patients
Bayer (2014)	Interventional	Unrelated to review question
Muzaffar (2013)	Interventional	Disease other than lymphoma
Henrich (2012)	Interventional	Less than 10 patients
Michieli (2011)	Expert Review	Study design
Marturano (2011)	Interventional	Primary CNS Lymphoma
Krishnan (2010)	Expert Review	Study design
Kaplan (2010)	Interventional	Does not include people with HIV
Krishnan (2009)	Expert Review	Study design
Krishnan (2003)	Expert Review	Study design
Kentos (2002)	Prospective	Disease other than lymphoma
Molina (2000)	Interventional	Less than 10 patients
Klionsky (2021)	Expert Review	Unrelated to review question
Goldman (2021)	Expert Review	Unrelated to review question
Carbone (2021)	Systematic Review	Study design
Noy (2020)	Expert Review	Study design

Kaplan (2012)	Expert Review	Study design
Ruhl (2009)	Expert Review	Study design
Carbone (2014)	Expert Review	Study design
Bociek (2005)	Expert Review	Study design
Biernat (2021)	Expert Review	Unrelated to review question
Szlasa (2022)	Expert Review	Unrelated to review question
Martis (2012)	Expert Review	Study design
Wagner-Johnston (2008)	Expert review	Study design
Smith (1996)	Expert review	Study design
Jantunen (2011)	Expert review	Unrelated to review question
Damon (2009)	Expert review	Unrelated to review question
Fuhr (2023)	Case Report	Unrelated to review question
Behler (2006)	Expert Review	Study design
Ambinder (2009)	Expert Review	Study design
Oertel (2002)	Expert Review	Unrelated to review question
Evens (2002)	Expert Review	Study design
Blair (2016)	Expert Review	Unrelated to review question
Stefan (2014)	Expert Review	Children included
Delagreverie (2019)	Retrospective	Did not report on our study outcomes
Krishnan (2002)	Expert Review	Study design
Spurgeon (2017)	Expert Review	Unrelated to review question
Molina (2000)	Expert Review	Study design
Hentrich (2014)	Systematic Review	Study design
Zanussi (2015)	Retrospective	Did not report on our study outcomes
Konishi (2022)	Prospective	Does not include people with HIV
Shindiapina (2021)	Interventional	Did not report on our study outcomes
Cashen (2009)	Expert Review	Unrelated to review question
Liu (2021)	Prospective	Did not report on our study outcomes
Zanet (2016)	Expert Review	Study design

Krishnan (2001)	Prospective	Less than 10 patients
Min (2018)	Case Report	Study design
Ross (2021)	Expert Review	Unrelated to review question
Pratesi (2015)	Prospective	Did not report on our study outcomes
Ketley (1997)	Expert Review	Unrelated to review question
Benicchi (2005)	Prospective	Less than 10 patients
Thiele (2021)	Retrospective	Did not report on our study outcomes
Hoffman (2016)	Prospective	Did not report on our study outcomes
DeClercq (2010)	Expert Review	Unrelated to our review question
Wieters (2014)	Retrospective	Less than 10 patients
Swinnen (2001)	Expert Review	Unrelated to our review question
Cheung (2005)	Expert Review	Study design
Xu (2020)	Case Report	Study design
Bertoli (2016)	Prospective	Did not report on our study outcomes
Marti-Carvajal (2007)	Systematic Review	Study design
Ahmed (2013)	Expert Review	Study design
Cillo (2013)	Prospective	Did not report on our study outcomes
Uy (2008)	Expert Review	Unrelated to review question
Danylesko (2016)	Prospective	Does not include people with HIV
Blade (1998)	Expert Review	Unrelated to review question
Keida (2004)	Case Report	Unrelated to review question
Junfeng (2019)	Case Report	Study design
Levine (2008)	Expert Review	Study design
Prakash (2022)	Prospective	Disease other than lymphoma
Okubo (2021)	Retrospective	Does not include people with HIV
Mert (2021)	Retrospective	Intervention other than ASCT
Zanelli (2021)	Systematic Review	Unrelated to review question
Valtola (2015)	Prospective	Does not include people with HIV
Ayala (2011)	Expert Review	Study design

Ferreri (2021)	Interventional	Intervention other than ASCT
Henrich (2017)	Prospective	Did not report on our study outcomes
Heyrman (2016)	Case Report	Unrelated to review question
Simonelli (2010)	Prospective	Did not report on our study outcomes
Cillo (2014)	Prospective	Intervention other than ASCT
Mounier (2007)	Expert Review	Study design
Hutter (2011)	Expert Review	Unrelated to review question
Emir (2014)	Retrospective	Includes children
King (2002)	Expert Review	Unrelated to review question
Spina (2003)	Expert Review	Study design
Ferreri (2017)	Prospective	Intervention other than ASCT
Mardani (2021)	Case Report	Study design
Nagai (2009)	Case Report	Study design
Henrich (2014)	Case Report	Study design
Rahav (2021)	Prospective	Unrelated to review question
Pusic (2008)	Prospective	Does not include people with HIV
Milone (2013)	Retrospective	Unrelated to review question
Malard (2012)	Prospective	Does not include people with HIV
Shaughnessy (2013)	Prospective	Does not include people with HIV
Costa (2011)	Prospective	Does not include people with HIV
Sanchez-Ortega (2015)	Retrospective	Does not include people with HIV
Varmavuo (2012)	Prospective	Unrelated to review question
Hagiwara (2013)	Prospective	Unrelated to review question
Hubel (2012)	Prospective	Does not include people with HIV
Wolach (2010)	Retrospective	Unrelated to review question
Savsek (2016)	Case Report	Unrelated to review question
Onishi (2023)	Case Report	Unrelated to review question
Eum (2006)	Case Report	Unrelated to review question
Goto (2011)	Case Report	Study design

Fraint (2021)	Case Report	Includes children
Zahid (2014)	Case Report	Includes children
Varmavuo (2012)	Prospective	Unrelated to review question
Sdrimas (2020)	Case Report	Study design
Wiernik (2001)	Expert Review	Study design
Kanellopoulos (2018)	Case Report	Unrelated to review question
Attolico (2012)	Prospective	Does not include people with HIV
Duell (1997)	Retrospective	Does not include people with HIV
DePaoli (2006)	Expert Review	Unrelated to review question
Agostinelli (2016)	Prospective	Unrelated to review question
Khare (2001)	Expert Review	Unrelated to review question
Herbert (2014)	Interventional	Unrelated to review question
Kawabata (2009)	Case Report	Study design
Flomemberg (2005)	Expert Review	Unrelated to review question
Shiraishi (2010)	Preclinical Trial	Unrelated to review question
Pratesi (2008)	Prospective	Did not report on our study outcomes
Zafar (2022)	Case Report	Unrelated to review question
Basak (2011)	Retrospective	Unrelated to review question
Broccoli (2018)	Case Report	Does not include people with HIV
Matikas (2014)	Case Report	Unrelated to review question
Amend (2010)	Retrospective	Does not include people with HIV
Pelonisi (2008)	Case Report	Study design
Shallis (2019)	Expert Review	Study design
Pippi (2008)	Expert Review	Study design
Orenstein (2005)	Case Report	Unrelated to review question
Jafarizade (2021)	Case Report	Unrelated to review question
Ahluwalia (2020)	Case Report	Unrelated to review question
Waddington (2004)	Case Report	Study design
Nguyen (2018)	Case Report	Unrelated to review question

Raufi (2016)	Case Report	Unrelated to review question
Lim (2005)	Expert Review	Study design
Cashen (2008)	Interventional	Does not include people with HIV
Fluri (2007)	Case Report	Includes children
Nguyen (2003)	Case Report	Does not include people with HIV
Brink (1997)	Histological	Unrelated to review question
Roblot (2003)	Retrospective	Unrelated to review question
Campbell (1999)	Case Report	Study design
Fenaux (2001)	Expert Review	Unrelated to review question
Sarris (2000)	Interventional	Unrelated to review question
Bollee (2007)	Prospective	Unrelated to review question
Kosmas (2019)	Expert Review	Unrelated to review question
Schade (2014)	Retrospective	Unrelated to review question
Won (2006)	Case Report	Does not include people with HIV
Serrao (2019)	Case Report	Unrelated to review question
Gabarre (1996)	Case Report	Study design
Montalbetti (1999)	Case Report	Unrelated to review question
Jantunen (2012)	Retrospective	Unrelated to review question
Allred (2021)	Case Report	Unrelated to review question
Osorio (2002)	Case Report	Unrelated to review question
Tekgunduz (2012)	Prospective	Unrelated to review question
Guerrero (2019)	Case Report	Unrelated to review question
Auer (2000)	Expert Review	Unrelated to review question
Dawson (2007)	Case Report	Study design
Ferreri (2004)	Retrospective	Unrelated to review question
Fedele (2006)	Retrospective	Unrelated to review question
Chanan-Khan	Case Report	Study design
Vose (1996)	Interventional	Unrelated to review question
Bandera (2006)	Expert Review	Study design

Chamberlain (1997)	Prospective	Unrelated to review question
Chen (2020)	Preclinical Trial	Unrelated to review question
Hunter (2009)	Interventional	Unrelated to review question
Orentas (1998)	Expert Review	Unrelated to review question
Kirby (2019)	Case Report	Study design
Koo (2001)	Preclinical trial	Unrelated to review question
Borenstein (2007)	Case Report	Does not include people with HIV
Madsen (2020)	Study Protocol	Unrelated to review question
Hoffman (2006)	Case Report	Study design
DelGiudice (2005)	Expert Review	Unrelated to review question
Szwebel (2021)	Case Report	Unrelated to review question
Dalia (2014)	Retrospective	Unrelated to review question
Jonasch (2001)	Expert Review	Unrelated to review question
Albring (2024)	Preclinical Trial	Unrelated to review question
Baker (2007)	Case Report	Unrelated to review question
Markasz (2006)	Histological	Unrelated to review question
Simonelli (2004)	Case Report	Study design
Akyay (2011)	Case Report	Unrelated to review question
Bilgin (2013)	Retrospective	Did not report on our study outcomes
Pinkerton (2002)	Prospective	Includes children
Segall (1996)	Preclinical Trial	Unrelated to review question
Gorschluter (2007)	Retrospective	Disease other than lymphoma
Eghbali (2000)	Expert Review	Study Design
Keogan (1999)	Retrospective	Unrelated to review question
Fantry (2002)	Expert Review	Unrelated to review question
Kojima (2015)	Case Report	Study design
Polprasert (2011)	Case Report	Unrelated to review question
Miceli (2012)	Case Report	Study design
Wood (2013)	Retrospective	Does not include people with HIV

Perram (2014)	Case Report	Unrelated to review question
Krishnan (2002)	Expert Review	Intervention other than ASCT
Haverkos (2014)	Prospective	Less than 10 patients
Nimmagadda (2010)	Case Report	Unrelated to review question
Schroeder (2009)	Case Report	Unrelated to review question
Prescott (1997)	Expert Review	Study design
Marr (1997)	Case Report	Unrelated to review question
Leclercq (1999)	Prospective	Did not report on our study outcomes
Nomura (2005)	Case Report	Unrelated to review question
Hecht (2002)	Prospective	Unrelated to review question
Barouch (2002)	Case Report	Unrelated to review question
Chowdhary (2008)	Case Report	Unrelated to review question
Fantry (2003)	Expert Review	Unrelated to review question
DiPersio (2012)	Expert Review	Unrelated to review question
Check (2005)	Expert Review	Unrelated to review question
Zorger (2025)	Systematic Review	Unrelated to review question
Hirsch (2025)	Systematic Review	Unrelated to review question
Kazmi (2025)	Systematic Review	Mixed disease types
Okada (2025)	Systematic Review	Study design
Okada (2025)	Systematic Review	Study design
Zanet (2025)	Retrospective	Study design
Shindiapina (2021)	Interventional	Did not report on our study outcomes
Yokota (2024)	Case Study	Study design
Lauriente (2024)	Case Study	Does not include people with HIV
Percic (2024)	Case Study	Disease other than lymphoma
Thomas (2025)	Semi-Structured Interviews	Unrelated to review question
de Goes	Case Report	Does not include people with HIV
Musheer (2026)	Systematic Review	Study design

Molla (2024)	Retrospective	Less than 10 patients
Mittal (2026)	Case Review	Unrelated to review question
Peterson (2024)	Retrospective	Conference Abstract
Qureshi (2024)	Retrospective	Conference Abstract
Martin (2024)	Retrospective	Conference Abstract
Huang (2024)	Retrospective	Conference Abstract
Comelles (2024)	Systematic Review	Conference Abstract
Giri Raja Pandiyan (2025)	Case Series	Less than 10 patients
Kiss (2025)	Interventional	Includes children
Yang (2024)	Case Report	Unrelated to review question
Sharp (2024)	Retrospective	Unrelated to review question
Amal (2025)	Case Report	Oral Conference Presentation
Vijenthira (2026)	Interventional	Intervention other than ASCT
Olafimihan (2025)	Retrospective	Includes children
Oganesyan (2025)	Systematic Review	Unrelated to review question
Teklie (2025)	Retrospective	Primary CNS Lymphoma
Dave (2024)	Retrospective	Primary CNS Lymphoma
Martin-Moro (2025)	Retrospective	Unrelated to review question
Alshehri (2024)	Retrospective	Includes children
Alderuccio (2024)	Retrospective	Unrelated to review question
Idoate (2024)	Retrospective	Did not report on our study outcomes
