# Supplementary material for: Autologous Stem Cell Transplant for HIV-Associated Lymphoma: A Systematic Review and Meta-Analysis
Source: Cancers (Basel). 2026 Jul 23;18(15):2373. doi: 10.3390/cancers18152373 (PMC13464129; doi:10.3390/cancers18152373)
Supplement: Supplementary file 1 [file cancers-18-02373-s001.zip › Table S3_JBI appraisal assessment_HIV_ASCTJuly14th.pdf]

Table S3. JBI critical appraisal of included quasi-experimental studies

Study	Q1 Cause/ effect clear	Q2 Control group	Q3 Groups similar	Q4 Similar care apart from intervention	Q5 Multiple outcome measurements	Q6 Outcomes measured same way	Q7 Outcomes measured reliably	Q8 Complete follow-up	Q9 Appropriate statistical analysis	Overall risk of bias (%)
**Alvarnas et al., 2016**	Y	Y	Y	U	Y	U	Y	Y	Y	Moderate (77.78)
**Krishnan et al., 2005**	Y	N	NA	NA	Y	NA	Y	Y	Y	Moderate (55.55)
**Re et al., 2009**	Y	N	NA	NA	Y	NA	Y	Y	Y	Moderate (55.55)
**Re et al., 2018**	Y	N	NA	NA	Y	NA	Y	Y	Y	Moderate (55.55)
**Serrano et al., 2005**	Y	N	NA	NA	Y	NA	Y	Y	Y	Moderate (55.55)
**Spitzer et al., 2008**	Y	N	NA	NA	Y	NA	Y	Y	Y	Moderate (55.55)

Y: Yes, N: No, U: Unclear, NA: Not applicable

Internal Validity	Alvarnas et al.	Krishnan et al.	Re et al. 2009	Re et al. 2018	Serrano et al.	Spitzer et al.
**Q1 Cause/effect clear**	Y	Y	Y	Y	Y	Y
**Q2 Control group**	Y	N	N	N	N	N
**Q3 Groups similar**	Y	NA	NA	NA	NA	NA
**Q4 Similar care apart from intervention**	U	NA	NA	NA	NA	NA
**Q5 Multiple outcome measurements**						
NRM	NA	NA	NA	NA	NA	NA
OS	Y	Y	Y	Y	Y	Y
PFS	Y	Y	Y	Y	Y	Y
**Q6 Outcomes measured same way**						
NRM	U	NA	NA	NA	NA	NA
OS	U	NA	NA	NA	NA	NA
PFS	U	NA	NA	NA	NA	NA
**Q7 Outcomes measured reliably**						
NRM	Y	Y	Y	Y	Y	Y
OS	Y	Y	Y	Y	Y	Y
PFS	Y	Y	Y	Y	Y	Y
**Q8 Complete follow-up**						
NRM	Y	Y	Y	Y	Y	Y
OS	Y	Y	Y	Y	Y	Y
PFS	Y	Y	Y	Y	Y	Y
**Q9 Appropriate statistical analysis**						
NRM	Y	Y	Y	Y	Y	Y
OS	Y	Y	Y	Y	Y	Y
PFS	Y	Y	Y	Y	Y	Y
**Overall risk of bias (%)**	**Moderate**	**Moderate**	**Moderate**	**Moderate**	**Moderate**	**Moderate**

NRM: Non-relapse mortality; OS: Overall survival; PFS: Progression-free survival; Y: Yes; N: No; U: Unclear; NA; Not applicable
